# Binding of Phenazinium Dye Safranin T to Polyriboadenylic Acid: Spectroscopic and Thermodynamic Study

**DOI:** 10.1371/journal.pone.0087992

**Published:** 2014-02-03

**Authors:** Ankur Bikash Pradhan, Lucy Haque, Snigdha Roy, Suman Das

**Affiliations:** Department of Chemistry, Jadavpur University, Jadavpur, Kolkata, India; Russian Academy of Sciences, Institute for Biological Instrumentation, Russian Federation

## Abstract

Here, we report results from experiments designed to explore the association of the phenazinium dye safranin T (ST, 3,7-diamino-2,8-dimethyl-5-phenylphenazinium chloride) with single and double stranded form of polyriboadenylic acid (hereafter poly-A) using several spectroscopic techniques. We demonstrate that the dye binds to single stranded polyriboadenylic acid (hereafter ss poly-A) with high affinity while it does not interact at all with the double stranded (ds) form of the polynucleotide. Fluorescence and absorption spectral studies reveal the molecular aspects of binding of ST to single stranded form of the polynucleotide. This observation is also supported by the circular dichroism study. Thermodynamic data obtained from temperature dependence of binding constant reveals that association is driven by negative enthalpy change and opposed by negative entropy change. Ferrocyanide quenching studies have shown intercalative binding of ST to ss poly-A. Experiments on viscosity measurements confirm the binding mode of the dye to be intercalative. The effect of [Na**^+^**] ion concentration on the binding process suggests the role of electrostatic forces in the complexation. Present studies reveal the utility of the dye in probing nucleic acid structure.

## Introduction

The use of small molecules to specifically control important cellular functions through binding to nucleic acids is an area of active interest at the interface of chemical biology and medicinal chemistry. The characterization of interaction of small molecules with nucleic acid not only provides insights in biology but also gives the opportunity for developing effective therapeutic agents [1], [2]. Some small molecules have been proven to be useful as sensitive probes for nucleic acid structures [3]. Since structural polymorphism of nucleic acids *in vivo* presents numerous opportunities for the development of new therapeutic agents, the design of small molecules that bind to noncanonical nucleic acid structures represents an active area of rational drug design [3]–[5]. The role of RNA in the progression of many diseases particularly in viral infections like HIV, AIDS and hepatitis B has led to growing interest in RNA as a potential target for therapeutic intervention [6]–[8]. Hence, the study of interaction between ligands with different polymorphic forms of RNA is not only of immense importance to understand the basic fundamentals of RNA-ligand interaction, but it is a necessity for the development of RNA targeted therapeutic agents.

Among the single stranded nucleic acids, polyriboadenylic acid [poly-A] is of particular biological relevance due to its role in mRNA functioning and gene expression [9], [10]. Single stranded poly-A has critical roles in cell biology [11], [12]. So it is important to understand how nucleic acid-targeting ligands affect such structures. Virtually all mRNAs in eukaryotic cells have a poly-A tail at the 3′ end that is an important determinant in the maturation and stability of mRNA and also the initiation of translation as well as the production of alternate forms of proteins [13]–[15]. Molecules that can interact with the polyadenylate tail may inhibit mRNA function and impair protein production in the cell. This switching off of the protein production by targeting poly-A tail may be an avenue for the development of RNA based therapeutic agents. Since the discovery that Neo-PAP (a recently identified human poly-A polymerase) is significantly over expressed in some human cancer cells [16], [17], it has been suggested that the poly-A tails of mRNA might interfere with the full processing of mRNA by PAP and switch off protein synthesis. This suggests that poly-A tail is a potential tumor-specific target [18]. Further, poly-A has been reported to exist in single stranded helical structure and parallel double stranded helix depending on a narrow pH variation and temperature [18]–[22]. Such a polymorphic conversion of poly-A makes it a prospective target for investigation to understand the binding and structural aspects through ligand interaction [23]–[27]. Polyadenylation process also plays a significant regulatory role in the production of alternative forms of proteins [28], [29]. A possible biological role for ds poly-A structure has been proposed by Zarudnaya et al. [30] and other group [31]. They have suggested the involvement of such structure in intracellular process as termination of mRNA–poly-A synthesis and auto regulation of poly-A binding protein synthesis.

**Figure 1:**
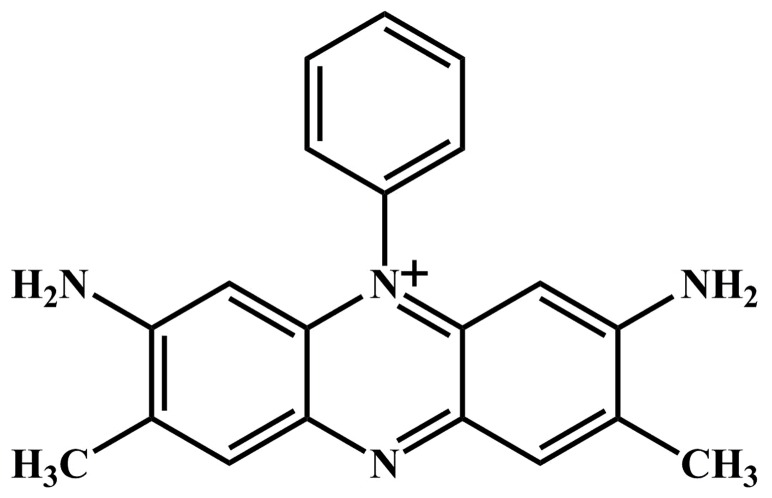
Chemical structure of Safranin T (ST).

Phenazine derivatives are a kind of antibiotic. Studies have shown that some phenazinium dyes have antimalarial potency and selectivity and they also inhibit many bacteria from growing [32]. Safranin T is a water soluble phenazinium dye which features a planar phenazine ring and a positive charge (Figure 1). This compound is mainly used as food dye in flavoring and colouring candies and cookies. It is also used for dyeing tannin, cotton, bast fibers, wool, silk, leather and paper [33]. A very wide range of biological application of ST has been reported [34], [35].

DNA binding aspects of ST has been reported and it has been shown that this ligand binds to DNA by the mechanism of intercalation [36]–[38]. Binding studies of this compound with RNA are scarce [39]. Due to the relevance of poly-A to mRNA stability, protein synthesis, virology, as well as the potential importance of poly-A to cancer biology, we choose to assess the ability of ST to target poly-A. We investigated in details the binding of ST to poly-A and our studies reveal its preferential binding to ss poly-A structure compared to the ds poly-A. This present study would be a good addition to the literature to understand the fundamentals of small molecule interactions with various RNA structures in order to develop RNA targeted therapeutic agents.

## Materials and Methods

### Biochemicals

Poly-A was a product from Sigma-Aldrich Corporation (St. Louis, MO, USA). Concentration of poly-A in terms of nucleotide was determined by UV absorbance measurements at 257 nm using molar extinction coefficient values of 10,000 M^−1^cm^−1^ [27]. Average molar mass of the poly-A used in our study was 2–2.7×10^5^ Da as determined from viscometric measurements. ST was purchased from Fluka (USA). Purity of ST was verified by thin layer chromatography and melting point determination [40]. Since no impurities were seen, no further purification was done.

### Preparation of stock solutions

ST solution was prepared freshly each day; the concentration of the compound was measured using molar extinction coefficients of 29,000 M^−1^ cm^−1^ at 520 nm. The compound obeyed Beer's law in the concentration range employed in the study. All the studies with ss poly-A were performed in Citrate-Phosphate (CP) buffer, pH 7.0 containing 5 mM of disodium hydrogen phosphate and 0.75 mM of citric acid. Salt dependent studies were performed in the same buffer containing different concentrations of Na^+^ ion.

The double stranded form of poly-A was prepared by very slow addition of ss poly-A solution into CP buffer of pH 4.5 under constant stirring and allowing three hour incubation at room temperature for the transition to be completed [27]. The formation of ds poly-A was verified by UV spectroscopy and circular dichroism (CD) study. Deionized and triple distilled water were used throughout our studies. All the buffer solutions were filtered through Millipore membrane filter of 0.45 µm before use.

### Absorption spectrophotometric measurements

All the UV-VIS absorption studies were made on a Shimadzu model UV-1800 spectrophotometer (Shimadzu Corporation, Japan) in matched quartz cells of 1 cm path length. A thermo programmer was attached to it to maintain the temperature of the spectrometer by Peltier effect. Spectrophotometric titrations were performed by keeping fixed concentration of compound and varying the concentration of polynucleotide. The change in the absorption at the λ_max_ of the dye was noted at each P/D ratio (RNA nucleotide/dye molar ratio) till saturation was obtained. This spectrophotometric data were then converted to titration curve of absorbance *versus* P/D ratio. The concentrations of free and bound dye were calculated from the derived points of the graph.

### Spectrofluorimetric measurements

Steady state fluorescence measurements were done in Horiba Jobin Yvon Fluoromax-4 spectrofluorimeter which was attached to highly sensitive temperature controller. Measurements were done in fluorescence free quartz cell of 1 cm path length. A fixed concentration of the dye was titrated by increasing concentration of poly-A under constant stirring condition.

### Evaluation of binding parameters from spectroscopic results

The results of absorption and fluorescence titration of ST with ss poly-A were expressed in terms of Scatchard plots as r/C_f_ *versus* r [41]. All the Scatchard plots were nonlinear and showed negative slopes at low r values as observed in non-cooperative binding isotherms and hence were analyzed by excluded site model for non-linear non-cooperative ligand binding phenomenon using McGhee and von Hippel equation [42]. 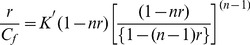
(1)


Here, K′ is the intrinsic binding constant to an isolated site; n is the number of nucleotides occluded after the binding of one single ligand molecule; r is the number of moles of ligand bound per mole of nucleotide and C_f_ is the molar concentration of free ligand. The binding data were analyzed using the Origin 7.0 software to determine the best-fit parameters of K′ and n.

### Determination of fluorescence polarization anisotropy

The steady state fluorescence anisotropy was also measured using the same spectrofluorimeter. Steady state anisotropy (r′), was defined by [43], 
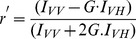
(2)


Where, G is the ratio I_HV_/I_HH_ used for instrumental correction. I_VV_, I_VH_, I_HV_ and I_HH_ represent the fluorescence signal for excitation and emission with the polarizer positions set at (0°,0°), (0°,90°), (90°, 0°) and (90°,90°), respectively.

### Fluorescence lifetime measurements

Time correlated single photon counting (TCSPC) measurements were carried out in 10 mM CP buffer of pH 7.0 at 20°C for the fluorescence decay of ST in the absence and in the presence of increasing concentration of poly-A. For the TCSPC measurements the photoexcitation was made at 450 nm using a picosecond diode laser (IBH Nanoled-07) in an IBH fluorocube apparatus. The fluorescence decay data were collected on a Hamamatsu MCP photo multiplier (R3809) and were analyzed by using IBH DAS6 software using the equation, 
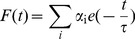
(3)where, α_i_ is the i^th^ preexponential factor and τ is the decay time. The decay time is life time of the excited species. The quality of fits was judged from χ^2^ criterion and visual inspection of the residuals of the fitted function to the data.

### Mode of binding: fluorescence quenching studies

Quenching studies were carried out with the anionic quencher potassium ferrocyanide. Solutions of KCl and K_4_[Fe(CN)_6_] were mixed in different ratios to give a fixed total ionic strength. Fluorescence quenching experiments were performed at a constant P/D ratio and fluorescence intensity was measured as a function of changing concentration of the ferrocyanide [44]. The data were plotted as Stern-Volmer plot of relative fluorescence intensity (*F*_o_/*F*) *versus* [Fe(CN)_6_]^4−^ using the equation [45], 
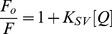
(4)where, *F*_o_ and *F* are the fluorescence intensities in the absence and in the presence of the quencher (Q) K_4_[Fe(CN)_6_]; K_SV_ is the Stern Volmer constant.

### Circular dichroic titration

Circular dichroism (CD) measurements were carried out on a PC-driven JASCO J815 spectropolarimeter (Jasco International Co.) attached with a temperature controller and a thermal programmer (model PFD-425L/15) interfaced in a rectangular quartz cuvette of 1 cm path length. All CD spectra were recorded in the wavelength range of 200–600 nm with a scan speed of 100 nm min^−1^. Each spectrum was averaged from five readings for each sample. Final CD spectra were expressed in terms of molar ellipticity ([θ]) in unit of deg cm^2^ dmol^−1^ by using the software provided with the spectropolarimeter. The molar ellipticity is based on nucleic acid concentration for the intrinsic CD and dye concentration for the extrinsic CD. All measurements were done at 20°C.

Influence of increasing ionic strength on the perturbation of ss poly-A CD spectrum by ST was investigated at four different salt concentrations, viz. 5, 10, 50 and 100 mM [Na^+^].

### Denaturation and renaturation studies

The denaturation and renaturation profiles of ss poly-A in the absence and in the presence of ST were taken at a rate of 0.5°C min^−1^ in the temperature range of 15°C to 60°C. Thermal melting profile of ds poly-A was taken in the temperature range 20°C to 110°C at the same rate of heating.

### Solution viscosity measurements

Viscometric measurements were carried out using a Cannon-Manning semi micro dilution viscometer type 75 (Cannon Instruments Co., State College, PA, USA) submerged vertically in a constant temperature bath maintained at 20±0.5°C. The molecular weight of the ss poly-A sample was estimated to be in the order 2–2.7×10^5^ Da with an intrinsic viscosity of 2.8 dL/g. 700 µL of RNA solution (500 µM for ss poly-A and 400 µM for ds poly-A) was placed in the viscometer and aliquots of stock solution of ST were directly added into the viscometer to obtain increasing D/P (ST/nucleotide molar ratio) values. Flow times of ss poly-A and ds poly-A in absence and in presence of increasing concentration of ST were measured in triplicate with an accuracy of ±0.01 s and the relative specific viscosity was calculated using the equation: 
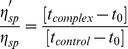
(5)where *η*'_sp_ and *η*_sp_ were the specific viscosity of ss poly-A or ds poly-A in presence and in absence of ST; *t*_complex_ and *t*_control_ were the time of flow of complex and control solution and *t*_0_ is the same for buffer solution as described previously (44).

The relative increase in length L/Lo were obtained from the corresponding increase in relative viscosity by using the following equation [46]. 
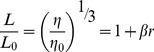
(6)


Here, L and Lo are the contour lengths of RNA in presence and absence of the dye, η and η_0_ are the corresponding values of intrinsic viscosity (approximated by the reduced viscosity η = η_sp_/C, where C is the RNA concentration and β is the slope when L/Lo is plotted against r.

### Temperature dependent spectrophotometric study

Temperature dependent absorption spectra were recorded by using Shimadzu UV-1800 of spectrophotometer attached with thermometric cell temperature programmer and temperature controller. These measurements were performed at 20, 25, 30 and 35°C allowing an equilibrium period of 15 minutes for each addition.

### Evaluation of thermodynamic parameters

The values of K′ and n were determined at different temperatures. Thermodynamic parameters were estimated by analysis of van't Hoff plot (ln K′ vs. 1/T) obtained over the temperature range of the study. The slope of the plot gives the binding enthalpy change (Δ*H* ^0^) as 
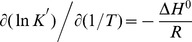
(7)


The Gibbs free energy change (Δ*G* ^0^) was determined from the binding constant at a particular temperature according to this relation 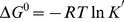
(8)


The entropy change (ΔS^0^) was estimated from the following relation 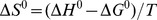
(9)


### Salt effect on binding

ST carries a positive charge and poly-A has a negative phosphate skeleton. Therefore in the binding process electrostatic contribution is thought to be one of the driving forces. Cations are condensed around the polyanionic poly-A helix and charged dye compete to expel the cations for phosphate neutralization; these are thermodynamically linked processes. To provide insight into such molecular details absorption and fluorimetric titrations were carried out at four different salt concentrations, viz. 5, 10, 25 and 50 mM [Na^+^], and the association constants (K′) were evaluated. The following relationship between K′ and [Na^+^] has been derived previously linking the charge to the variation of binding affinity with [Na**^+^**] [47]. 
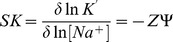
(10)


Here, Z is the apparent charge of the bound ligand per phosphate binding. Ψ is the fraction of the [Na^+^] bound per RNA phosphate and SK is equivalent to the number of counterions released upon binding of a dye. From the salt dependence of binding constant the electrostatic (*Δ*G*_pe_*) and nonelectrostatic (*ΔG_t_*) contributions to the overall Gibbs free energy was calculated. The polyelectrolytic contribution to the overall observed free energy can be quantitatively estimated from the relationship, 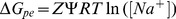
(11)


At a given salt concentration, the non electrostatic contribution can be calculated as the difference between *ΔG* and *Δ*G*_pe_*.

## Results and discussion

### Spectral characteristics of poly-A structure

**Figure 2:**
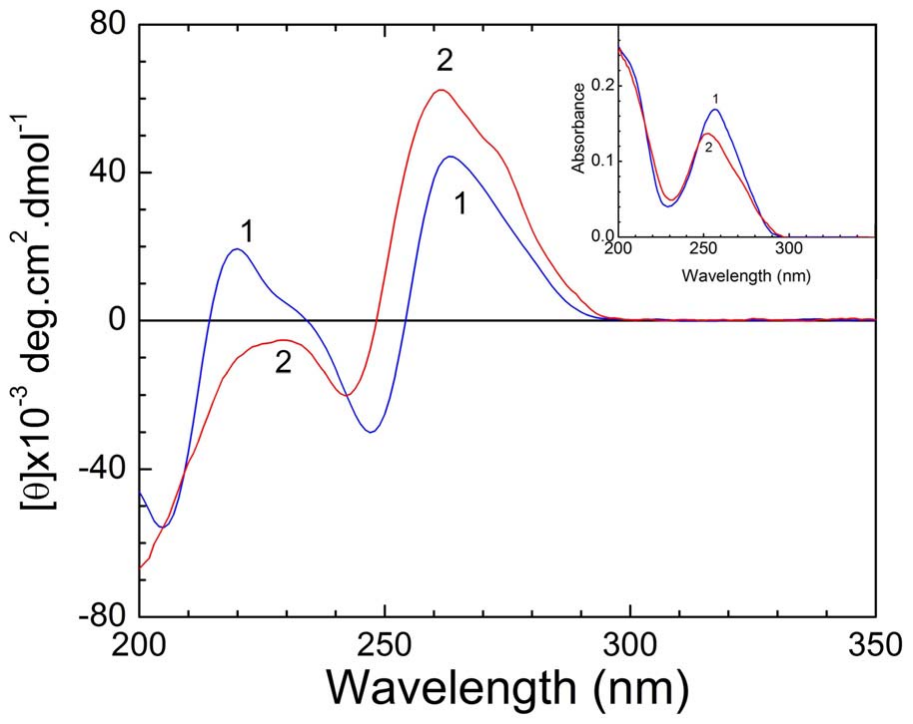
Circular Dichorism spectral titration of poly-A with ST. CD spectra of ss poly-A (17.0 µM, curve 1) and ds poly-A (17.0 µM, curve 2) at 20°C in 10 mM CP buffer, pH 7.0 and 4.5 respectively. Inset: UV spectra ss poly-A (17.0 µM, curve 1) and ds poly-A (17.0 µM, curve 2) at 20°C in 10 mM CP buffer, pH 7.0 and 4.5 respectively.

Characteristic circular dichroism (CD) spectra of poly-A at pH 7.0 and 4.5 for single stranded and double stranded structures are presented in Figure 2. Corresponding absorption spectra are shown in the inset. The CD spectrum of the ss poly-A structure at pH 7.0 has a positive band around 264 nm and a strong negative band around 248 nm followed by a positive band 220 nm. When pH was lowered to 4.5 the positive band was enhanced in ellipticity and was blue shifted while the negative band was blue shifted and became weaker with a peak at around 242 nm. The positive band around 220 nm in the ss poly-A structure was disappeared and a negative shoulder appeared around 234 nm. The absorption spectrum (inset of Figure 2) of ss poly-A at pH 7.0 had a maximum at 257 nm and at pH 4.5 this band maximum was blue shifted to 252 nm with a hypochromic effect. All these changes characterized the transition of poly-A from a single stranded helix to a double stranded helix [48].

### Binding characteristics of ST–poly-A interaction: Spectrophotometric titration

**Figure 3:**
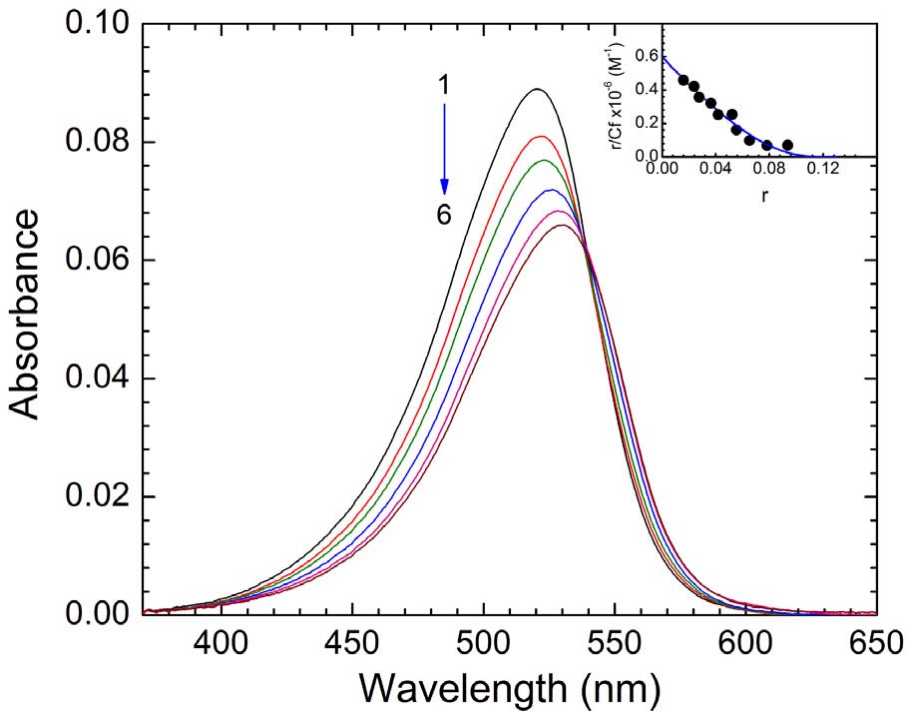
Absorption spectral change of ST in presence of poly-A. Representative absorption spectrum of ST (3.03 µM) treated with increasing concentration of ss poly-A in 10 mM CP buffer pH 7.0 at 20°C: Spectra 1–6 denote ss poly-A concentrations of 0, 12.02, 16.40, 26.02, 40 and 72.50 µM. Inset: Scatchard plot for the binding of ST to ss Poly-A. The solid line is the non linear least squares best fit of the experimental points to the Von Hippel equation.

The effect of increasing concentration of ss poly-A on the absorption spectrum of ST is presented in Figure 3. The interaction of the ST with ss poly-A resulted in marked changes in the visible region (370–650 nm) of the dye absorption spectrum with hypochromic and bathochromic effects that essentially indicated strong intermolecular interaction involving effective overlap of the π electron cloud of ST with the RNA bases. Pronounced hypochromic effect in the dye spectrum could be the indication of intercalative binding of ST between the bases of single stranded RNA. Presence of a sharp isosbestic point at 537 nm clearly indicates the equilibrium between bound and the free dye molecules [44]. Similar type of spectral features on the interaction of ST with calf thymus (CT) DNA have also been reported by Cao and He [36]. In their study two isosbestic points were observed at 432 nm and 538 nm respectively. In the present study on the interaction of ss poly-A we have observed only one isosbestic point.

The absorption titration data were expressed in the form of Scatchard plot (inset of Figure 3) which was observed to be nonlinear and noncooperative in nature. Therefore the Scatchard plot was fitted to a theoretical curve drawn according to the excluded site model developed by McGhee and von Hippel [42] for a nonlinear non-cooperative ligand binding. The best fit values of the binding parameters calculated from Equation 1 are presented in Table 1. The striking result that emerged from this experiment and analysis was that ST binds to ss poly-A in a noncooperative manner with an intrinsic binding affinity (K′) of 5.98×10^5^ M^−1^ and an exclusion site (n) of 7.4 bases (at 20°C, Table 1). Similar stoichiometry (∼7) was also obtained from continuous variation method (Job's plot) using both spectrofluorimetry and UV-vis spectrophotometry (data not shown). Binding of ST with CT DNA, fish sperm (FS) DNA and yeast RNA has been reported by Huang *et al* [39]. In our study on the interaction of ST with ss poly-A, binding constant has been found to be of the same order (∼10^5^ M^−1^) as that of the data reported by them (K′ = 1.25×10^5^, 2.40×10^5^ and 2.41×10^5^ M^−1^ with CT DNA, FS DNA and yeast RNA respectively). In comparison with the data reported by Huang *et al* on the binding of ST with CT DNA (n = 4.2) FS DNA (n = 4.3) and yeast RNA (n = 2.9), in our case the binding stoichiometry (n∼7) has been found to be large for ST-ss poly-A interaction. This may be attributed to the differences in the sequences of polymers used and their structures in the solutions. In an another study [36] the binding constant for the association of ST with CT DNA has been reported to less (∼10^4^ M^−1^). This may be due to the difference in the components, pH and ionic strength of the buffer solution used. In our study no effect of ds poly-A on the absorption spectrum of ST was noticed up to a *P/D* ratio of 100 (Figure S1, panel A). This confirmed the absence of any binding of ST with ds poly-A.

**Table 1:** Binding parameters for the interaction of ST with ss poly-A in 10 mM CP buffer, pH 7.0 at 20°C obtained from spectrophotometry and spectrofluorimetry^a^.

Parameters	Methods	Values
K′×10^−5^, the intrinsic binding constant (M^−1^)	[A] Spectrophotometry	5.98±0.20
	[B] Spectrofluorimetry	6.15±0.20
n, the no of base occluded	[A] Spectrophotometry	7.40±0.30
	[B] Spectrofluorimetry	7.60±0.20
K_SV_, the Stern Volmer quenching constant (L mol^−1^)	Spectrofluorimetry	[i] Free: 32.5±2.30 [ii] Bound: 8.0±0.70
Fluorescence polarization anisotropy	Spectrofluorimetry	[i] Free:0.030±0.002 [ii] Bound:0.18±0.01

a Average of three determinations.

### Fluorescence spectral study

**Figure 4:**
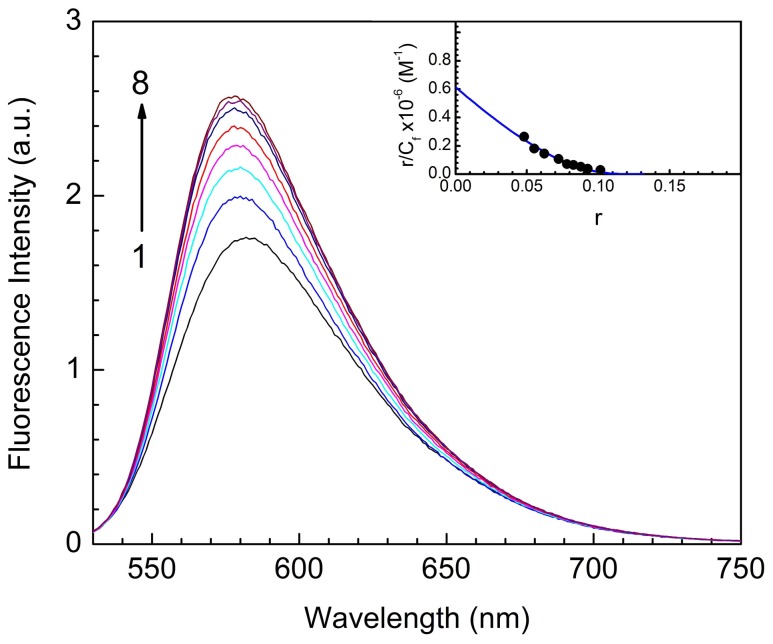
Fluorescence spectra of ST in presence of poly-A. Fluorescence spectra of ST in presence of ss poly-A in 10 mM CP buffer pH 7.0 at 20°C: Spectra 1–8 denote the fluorescence spectrum of ST (5.0 µM) treated with 0, 15.0, 27.0, 37.5, 50.0, 75.0, 100.0 and 125.0 µM of the ss poly-A. Inset: Scatchard plot for the binding of ST to ss poly-A. The solid line is the non linear least squares best fit of the experimental points to the Von Hippel equation.

ST is a fluorescent molecule. The emission spectrum of ST was recorded in the wavelength range 530–750 nm with maximum around 580 nm when excited at 520 nm. Complex formation was monitored by titration studies keeping a constant concentration of the dye and increasing the concentration of ss poly-A. With increasing concentration of ss poly-A a progressive enhancement in the fluorescence intensity of the dye was observed and eventually reaching a saturation point with approximately 3 nm blue shift in the wavelength maximum (Figure 4).The results of the spectrofluorimetric titrations were analyzed by constructing Scatchard plot. The Scatchard plot (inset of Figure 4) exhibited non-cooperative behavior as revealed by negative slope and hence were analyzed further by the McGheee and von Hippel methodology [42] for noncooperative binding using Equation 1 for the evaluation of the binding parameters. Analysis yielded a binding constant of 6.15×10^5^ M^−1^ and n value of 7.60 (at 20°C), which are in excellent agreement with the spectrophotometric results (table 1). The values of binding parameters are presented in the Table 1. Here also no effect of ds poly-A on the fluorescence spectrum of ST up to a P/D ratio of 100 confirmed the absence of binding of the dye to ds poly-A (Figure S1, panel B).

### Mode of binding: fluorescence quenching studies

**Figure 5:**
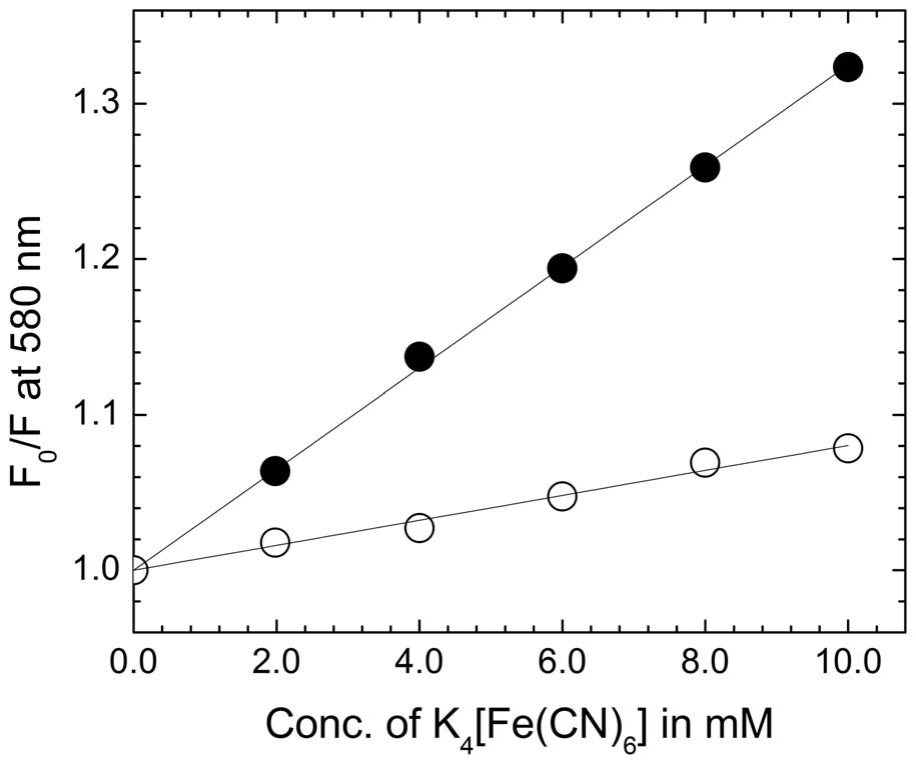
Stern–Volmer plots. Quenching of ST fluorescence by [Fe(CN)_6_]^4−^ at 20°C in the absence (•) and in presence (○) of ss poly-A in 10 mM CP buffer, pH 7.0.

Fluorescence quenching experiments provide an effective method to address the mode of binding of small ligands to nucleic acids [44]. In principle, molecules that are either free or bound on the surface of nucleic acid are easily accessible for the quencher while those are inserted between bases of the polynucleotide may not be accessible to the quencher. Negative charge on the phosphate backbone of the polynucleotide causes an electrostatic barrier at the helix surface and limits the penetration of an anionic quencher into the interior core of the helix. As a result very little or no quenching may be observed in the presence of such quencher if the binding involves strong stacking or intercalation and consequently the magnitude of the Stern-Volmer quenching constant (K_SV_) of the ligands that are bound inside will be lower than that of the free molecules. It is observed that binding to the poly-A resulted in an increase of the fluorescence intensity of ST (Figure 4). Representative Stern-Volmer plots for free and RNA bound ST are shown in Figure 5. K_SV_ values for free ST and its complex with ss poly-A were 32.5 and 8.0 L mol^−1^ respectively. This indicated that bound ST was less accessible to the quencher or in other words were considerably protected and sequestered away from the solvent suggesting intercalative binding with ss poly-A. Similar kind of results have also been reported for the binding of methylene blue, ethidium bromide, propidium iodide with ss poly-A [48], [49].

### Steady state fluorescence polarization anisotropy study

**Figure 6:**
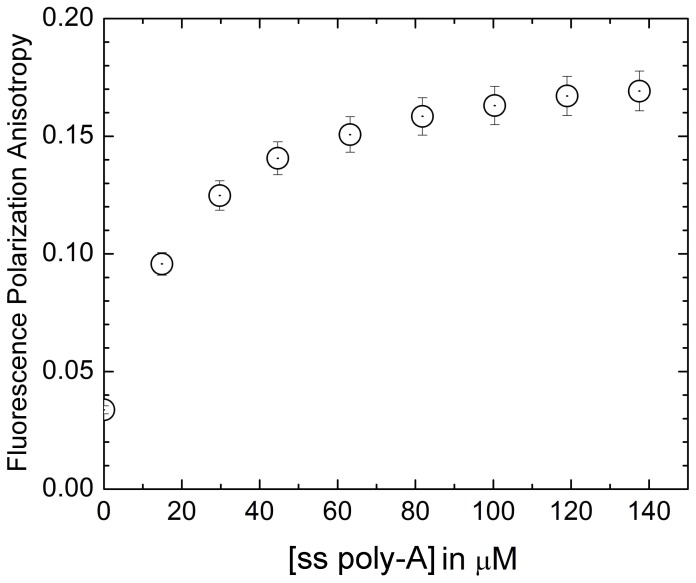
Variation of the anisotropy. Variation of the anisotropy of ST fluorescence as a function of concentration of ss poly-A. λ_ex_ and λ_em_ for ST was 520 nm and 580 nm respectively.

Fluorescence anisotropy measurements provide effective information about the nature of the environment of probes. Any factor that affects the shape, size and flexibility of a molecule alters the observed anisotropy [45]. Increase in the rigidity of the environment surrounding the fluorophore causes an increase in the fluorescence anisotropy. Change in anisotropy can thus help in finding the probable location of a probe in environments like nucleic acids [50]. Figure 6 shows the variation of fluorescence anisotropy with increasing concentration of ss poly-A for the fluorophore ST. A significant increase in the fluorescence anisotropy on binding with ss poly-A suggests that the dye was trapped in a motionally restricted region within the polynucleotide. It has been found that the fluorescence polarization anisotropy of ST upon binding to the ss poly-A showed a value of 0.18 at saturation against a value of 0.03 for free ST under identical condition. This increase in fluorescence polarization anisotropy provided insight towards strong interaction, probably intercalation, of ST to ss poly-A but further studies like quenching and viscosity measurements were done to illustrate the mode of binding.

### Lifetime measurements

**Figure 7:**
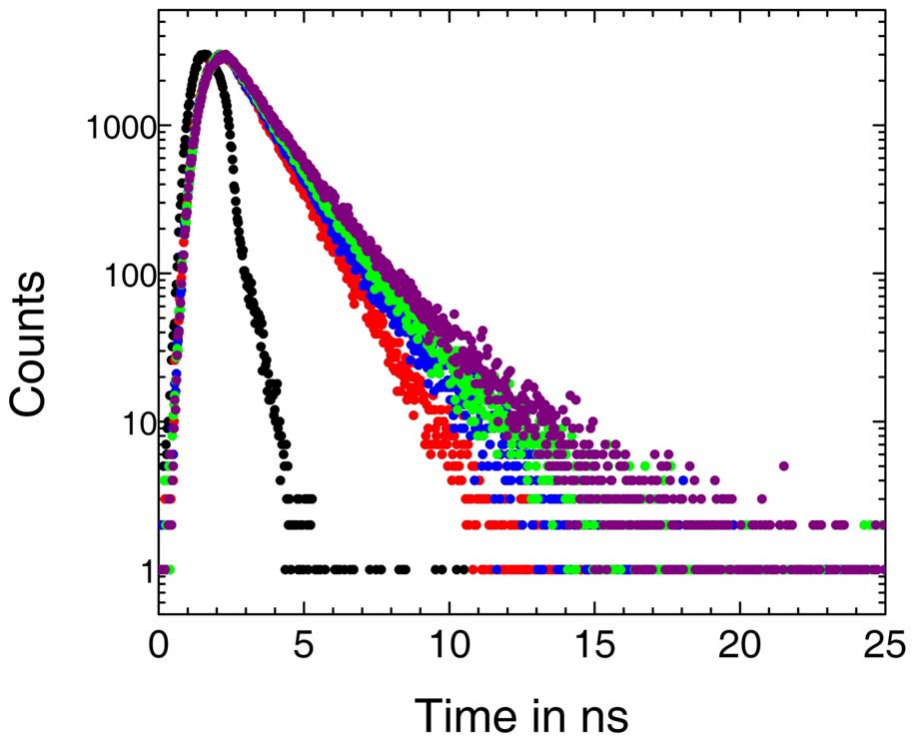
Time correlated single photon counting measurements. Time-resolved fluorescence decay curves (logarithm of normalised intensity versus time in ns) for ST (8.0 µM) in absence (red) and in presence of 40.0 (blue), 80.0 (green), 160.0 (purple) µM of ss poly-A at 20°C in 10 mM CP buffer of pH 7.0. (black) indicates decay curve for the scatterer.

The fluorescence decay profiles of ST in presence and in absence of ss poly-A are shown in Figure 7. The lifetimes obtained from the best fittings to the decay traces are presented in Table 2. Goodness of the fits has been evaluated from the *χ* ^2^ criterion (*χ* ^2^ within 1.0–1.1). It was observed that ST had monoexponential decays in absence as well as in presence of the added polynucleotide. With increase in concentration of ss poly-A lifetime increased gradually up to the saturating condition. Increase in values of lifetime showed strong binding of the compound to the polymer.

**Table 2:** Fluorescence lifetime of ST in absence and in presence of ss poly-A in 10 mM CP buffer, pH 7.0 at 20°C.

[Dye] µM	[Poly-A]/[Dye]	τ^a^ (ns)	χ^2^ b
8.58	0.00	1.09	1.06
8.58	8.4	1.37	1.09
8.58	16.8	1.52	1.09
8.58	33.6	1.95	1.03

a Life time of the excited species;

b Reduced chi-square.

### Circular dichroism spectral study

**Figure 8:**
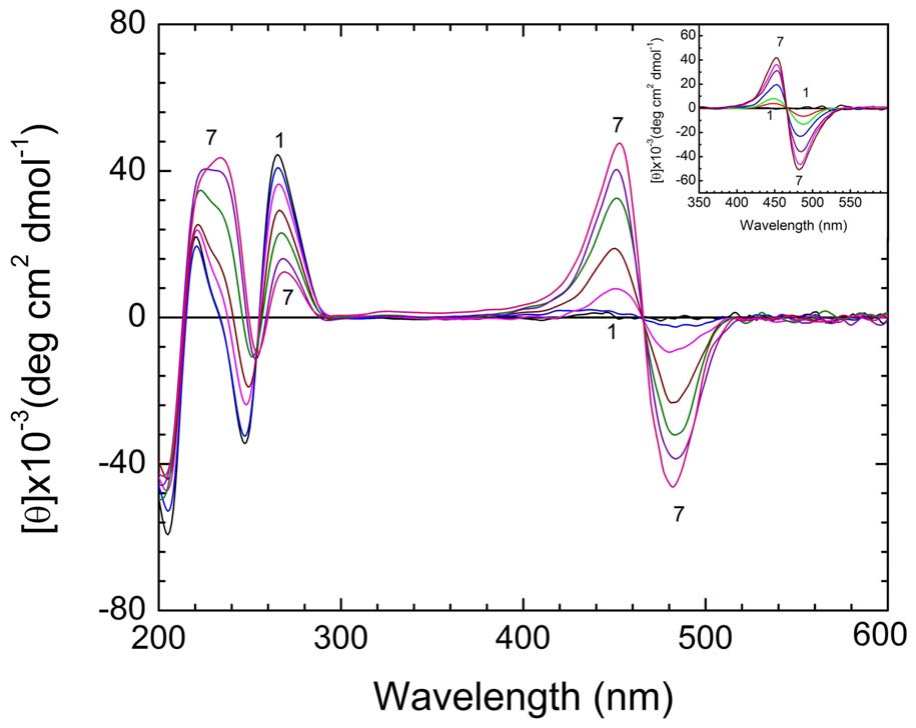
Circular dichroism spectral titration of poly-A with ST. Circular dichroic spectra of ss Poly-A (100.0 µM) with varying concentrations of ST in 10 mM CP buffer pH 7.0 at 20°C. The curves 1–7 represent the ST concentrations of 0, 4.28, 10.68, 21.32, 31.91, 42.44 and 52.92 µM respectively. Inset represents the induced CD spectra of (21.0 µM) of ST with increasing concentrations of ss poly-A. Curves 1–7 represent ss poly-A concentrations of 0, 38.3, 76.6, 152.8, 228.6, 305.2 and 380.2 µM respectively.

The CD spectrum of ss poly-A was characterized by a large positive band around 264 nm and a negative band around 248 nm. With increasing concentration of ST the CD spectral nature was strongly perturbed (Figure 8). The intensity of 264 nm band with an initial molar ellipticity of around 44,000 deg cm^2^ dmol^−1^ decreased when titrated with ST. In presence of the dye the peak was red shifted to around 269 nm and ellipticity became around 12,500 deg cm^2^ dmol^−1^. The characteristic negative CD band around 248 nm increased from an initial molar ellipticity of −35,000 deg cm^2^ dmol^−1^. Finally at saturation this peak was red shifted to 253 nm and ellipticity went upto −9,000 deg cm^2^ dmol^−1^. A clear isoelliptic point at 254 nm was observed for the ss poly-A-ligand interaction. ST molecule itself does not show any characteristic CD in its absorption range due to its molecular symmetry. Figure 8 shows that there is appearance of induced CD band in the absorption region of ST. This is due to the molecular asymmetry induced by the RNA polynucleotide on ST.

Inset of Figure 8 represents the induced CD of ST in presence of added single stranded polynucleotide. Here, to a fixed concentration of ST, ss poly-A was added until saturation was reached. In presence of added single stranded form of the polymer there was formation of induced CD band with enhanced ellipticity. These induced CD spectra occurred in a wavelength region where the RNA had no absorption band and thus exclusively monitored changes in the environment of the dye molecules. It is pertinent to note here that ST being achiral and planar is not CD active by itself. Therefore the induced CD spectrum was generated exclusively from the asymmetric arrangement of the dye that had been intercalated into the RNA bases. The presence of isoelliptic point at 466 nm in the series of spectra suggested that the bound and free molecules were in equilibrium as revealed from absorption spectral studies also. Appearance of such strong CD band in presence of the polynucleotide confirmed the intercalative mode of binding of ST with ss poly-A. Similar observation has been reported for ST binding with CT DNA [38]. No change in the CD spectrum of ds poly-A in presence of ST clearly indicated the absence of any interaction of ST with double stranded form of the polynucleotide (Figure S1, panel C). This supports our earlier observation in spectrophotometric and spectrofluorimetric data.

### CD melting study

The effect of temperature on ss poly-A structure in absence and in presence of the dye was monitored using CD spectroscopy (Figure S2). It was observed that ss poly-A went denaturation (structural transition from helix to random coil) with rise of temperature from 20 to 60°C and a complete renaturation occurred with lowering in temperature to 20°C. This clearly indicated a complete transition of the helical structure of ss poly-A to random coil structure and back. No change in molar ellipticity value of the bound form was observed on heating or cooling at D/P∼0.53 in the same temperature range as mentioned earlier. This indicates that the dye binds to ss poly-A. In our experimental conditions there was absence of any self-structure formation of poly-A in presence of ST. Similar kind of observation has also been reported for ethidium bromide and propidium iodide [48]. Thermal melting profiles of ds poly-A in absence and in presence of the dye are shown in panel C of Figure S2. Double helical to single helical transition was observed around 74°C. No change in the profile in presence of the dye again confirmed the absence of any binding of ST with the double stranded form of the polynucleotide.

### Viscometric study

**Figure 9:**
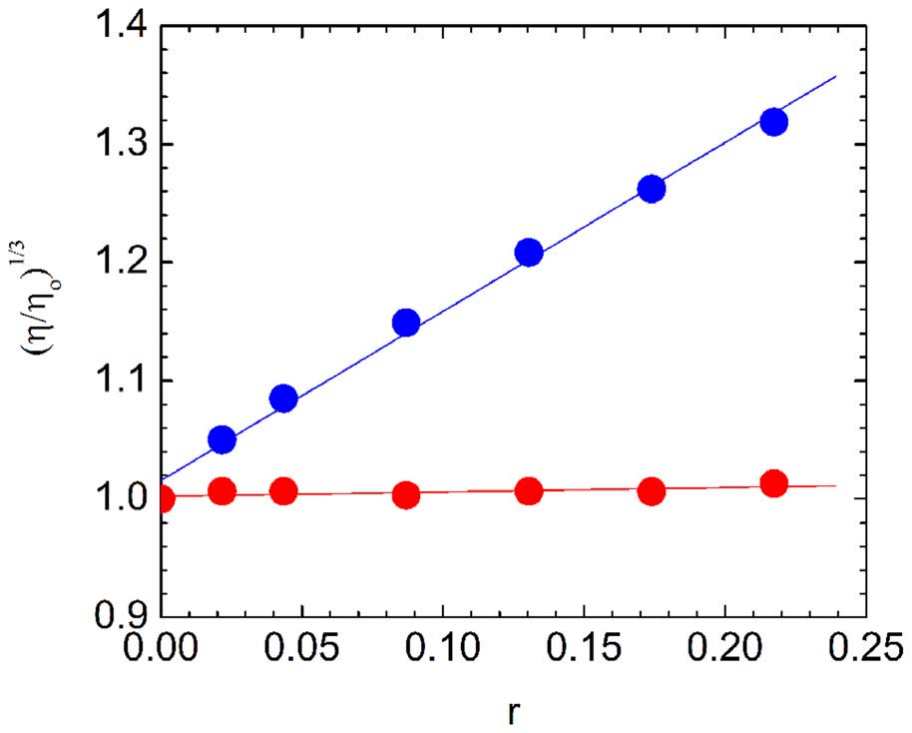
Viscometric measurements. Plot of change of relative specific viscosity of ss poly-A (blue) and ds poly-A (red) with increasing concentration of ST in 10 mM CP buffer pH 7.0 or 4.5 at 20°C. The concentrations of ss poly-A and ds poly-A were 500 and 400 µM, respectively.

The mode of binding by which ST interacts with poly-A was investigated by viscometric techniques. Hydrodynamic measurements are very sensitive to length changes and stiffening in rod like nucleic acid by insertion of ligands between adjacent bases [51] and are regarded as the most decisive and reliable test for elucidating the binding mode of small molecules to nucleic acids in solution. Ligand insertion between the bases is associated with the lengthening of the nucleic acid and this increase in length manifests itself as an increase in the viscosity of the nucleic acid solution. Thus ligand induced enhancement in the viscosity in the solution of nucleic acid is consistent with an intercalative mode of binding. On the other hand, the lack of a ligand induced increase in viscosity is consistent with a non-intercalative mode of binding (e.g. groove binding). In Figure 9, a plot of (η/η_0_)^1/3^ versus r for the complexation of ST to ss and ds poly-A is presented. Increase in viscosity of ss poly-A solution was observed in presence of ST while no change was noted in case of solution of ds poly-A. The value of the slope obtained for ss poly-A-ST interaction was ∼1.4 and this in turn lead to a helix length extension (ΔL) of 0.24 nm at the binding site calculated from a standard value of β = 2 corresponding to a length enhancement of 0.34 nm. This result clearly established intercalation type of binding of ST to ss poly-A helical structure. It is to be noted that, since ss poly-A does not have any base pairing and has only stacked helical structure, a true intercalation model where planar ligand molecules are fully sandwiched between hydrogen-bonded base pairs of double stranded DNA cannot be ideated.

### Thermodynamics of the interaction

**Figure 10:**
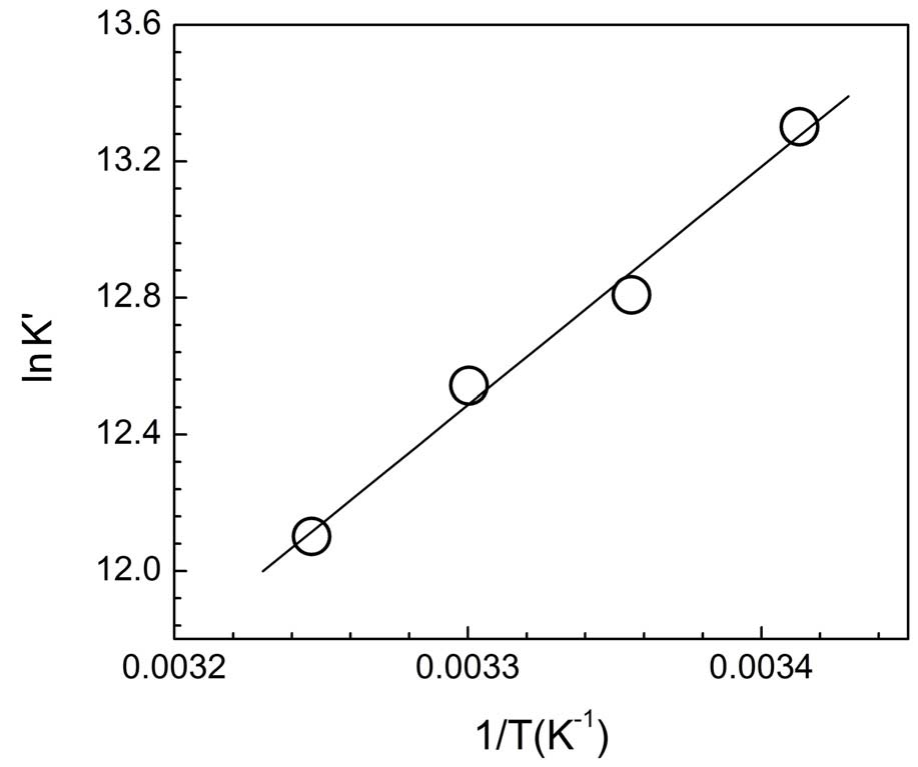
Temperature dependence of binding constant. Van't Hoff plot for the complexation of ST with ss poly-A in 10 mM CP buffer of pH 7.0. The data were fitted to straight line with correlation coefficient of 0.97.

Thermodynamic parameters for the association of ST with ss poly-A were calculated from the temperature dependence of binding constants using UV-Vis absorption spectroscopic studies at four different temperatures namely 20, 25, 30 and 35°C respectively at constant salt molarity of 10 mM [Na^+^].The binding parameters are presented in Table 3. It can be seen that with increase in temperature the binding constant decreased while the number of nucleotide occluded sites (n) changed marginally. The van't Hoff plot for binding is shown in Figure 10. Linear fit of the data indicates a very small value of heat capacity change (ΔC_P_≈0). The values of the thermodynamic parameters are presented in Table 3. It can be seen that the binding of ST with ss poly-A was characterized by both negative enthalpy and entropy changes.

**Table 3:** Binding and thermodynamic parameters for the interaction of ST with ss poly-A in 10 mM CP buffer, pH 7.0 obtained from absorption spectrophotometry^a^.

Temperature (°C)	K′×10^−5^(M^−1^)	n	ΔG°(kJ mol^−1^) at 20°C	ΔH° (kJ mol^−1^)	TΔS° (kJ mol^−1^) at 20°C
20	5.98±0.20	7.40±0.30	−32.39±2.0	−58.16±2.5	−25.77±0.10
25	3.30±0.20	7.60±0.32			
30	2.01±0.18	7.65±0.30			
35	0.95±10.12	7.70±0.35			

a Average of three determinations.

Conceptually the thermodynamic parameters for the binding process may be divided into three contributions: [i] contributions from hydrogen bonding and hydrophobic part due to interactions between the bound dye and nucleic acid binding site; [ii] contribution from the conformational changes upon binding in either the nucleic acid or the ligand and [iii] contributions from coupled processes like ion release, proton transfer or changes in the water of hydration. Our data revealed that the binding process was favoured by negative enthalpy change and was opposed by negative entropy change. The possible contribution of negative enthalpy change for the binding process of ST to ss poly-A may be explained in terms of the van der Waal's stacking interactions, hydrophobic as well as weak electrostatic interactions. Negative entropy change for the interaction process may be attributed to the increase in order due to the formation of rigid polynucleotide-dye complex.

### Dependence of the binding on ionic strength

**Figure 11:**
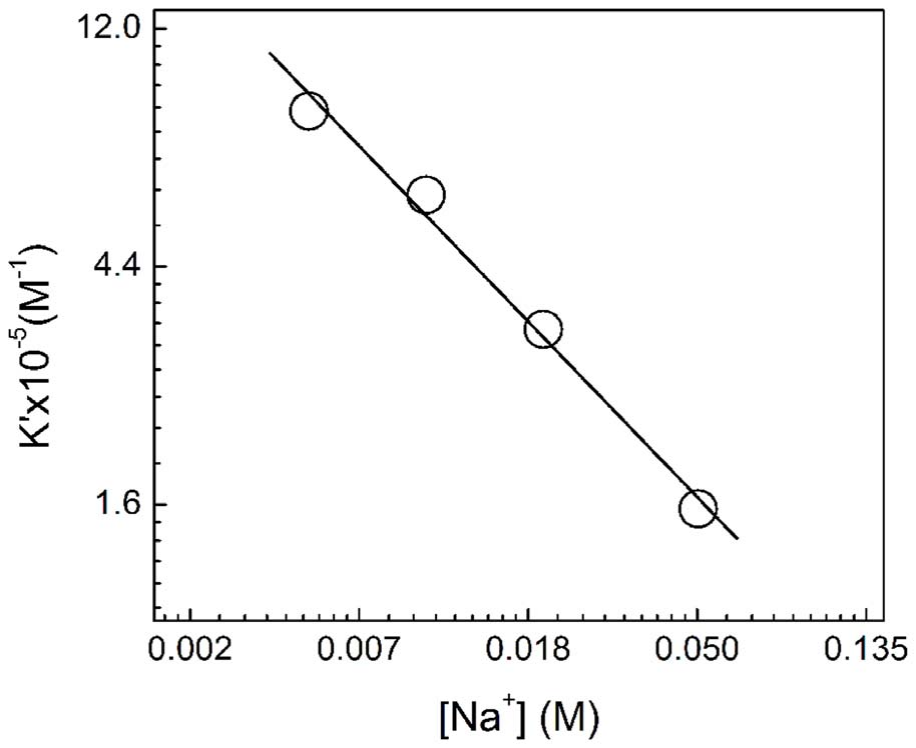
Salt dependence of binding constant. ln–ln plot of intrinsic binding constant (K′) as a function of ionic strength [Na**^+^**] for ST binding to ss poly-A in CP buffer of pH 7.0 at 20°C.

It has been observed that aromatic systems comprised of two condensed rings can enforce destacking of the nucleobases leading to intercalation only if their association to nucleic acid is facilitated by positive charges in the ring system. RNA condenses counter ions on its surfaces to screen the polyionic charge on the phosphodiester backbone. The phenazinium dye ST is a cationic molecule that may compete with sodium ions for binding to RNA phosphates. To assess the salt dependence of binding of ST with ss poly-A we have done binding studies using spectrophotometry at varying concentration of Na^+^. Variation of K′ versus [Na^+^] is plotted in Figure 11. Data on salt dependence of binding are presented in Table 4. The binding constants were found to decrease with increase of salt concentration. Here the plot was found to be linear with slope (−ZΨ) being −0.73. The physical meaning of this value is that the thermodynamic extent of counterion released from each phosphate on binding of single ST molecule is 0.73. Similar data have been reported for other cationic molecules binding to double stranded DNAs and RNAs [48], [52]. The salt dependence of binding clearly emphasized the role of the electrostatic component in the binding process. It has been observed that as the salt concentration was increased, the ΔG_pe_ contribution decreased while nonelectrolytic contribution remained almost unchanged. Data presented in Table 4 clearly revealed that there was a remarkably large contribution from the nonelectrostatic forces to the overall binding free energy. This clearly suggested the larger role of hydrophobic forces in the binding process of ST to ss poly-A. This is in good agreement with the report on cationic molecule binding to double stranded DNAs and RNAs [48], [52]–[54].

**Table 4:** Ionic strength dependence of binding constant for the interaction of ST with ss poly-A obtained from absorption spectrophotometry^a^.

[Na^+^] (M)	K′×10^−5^(M^−1^)	ZΨ	ΔG°(kJ mol^−1^) at 20°C		
			ΔG°	ΔG°t	ΔG°pe
0.005	8.50±0.25	0.73	−33.26±2.1	−23.85±1.4	−9.41±1.2
0.010	5.98±0.20		−32.39±2.0	−24.18±1.5	−8.21±1.0
0.020	3.40±0.20		−31.05±1.9	−24.1±1.5	−6.95±0.8
0.050	1.60±0.15		−29.21±1.6	−23.89±1.6	−5.32±0.6

a All values refer to solution conditions of pH 7.0 at 20°C. −ZΨ is the slope of the plot of lnK′ versus ln[Na+]. All other parameters are as defined in the text.

### Salt dependent CD studies

To understand the role of electrostatic interaction in the binding process, salt dependence of the binding of ST with ss poly-A was performed by CD experiment. The CD spectral changes of the interaction were followed at four Na^+^ concentrations viz. 05, 10, 20 and 50 mM to complement the observed decrease in binding as the salt increased. The data is presented in Figure S3. It can be seen that the conformational changes in poly-A were less pronounced as the salt concentration enhanced. This was also complemented by the lower intensity for the induced CD bands of ST in the complex. This result confirmed that the binding was favoured at lower salt concentration probably due to the fact that below 50 mM of [Na**^+^**] no self-assembled structure was formed in poly-A in presence of ST.

## Conclusion

The phenazinium dye has been shown to interact strongly with B-form DNA by the mechanism of intercalation [36]–[38]. A few studies have been reported on the interaction of phenazinium dye with RNA structures [39]. The strong interaction of ST with ss poly-A was evident from the bathochromic and hypochromic effect in the absorption spectra; significant enhancement of fluorescence intensity, fluorescence polarization anisotropy and life lime; remarkable perturbation of CD spectra; enhancement of viscosity of the polynucleotide solution and magnitude of thermodynamic parameters. From the data presented here it can be concluded that the compound binds to the single stranded form of the polynucleotide by the mechanism of intercalation while it does not bind at all with the double stranded form polyriboadenylic acid. CD melting study shows ST could not induce self structure formation in poly-A. In the binding process there was a remarkably large magnitude from the nonelectrostatic forces to the binding free energy which clearly suggested the larger role of hydrophobic forces in the binding process. The specific ss poly-A binding features of ST would turn into further opportunity for RNA based therapeutic agents.

## Supporting Information

Figure S1 **Characterization of interaction of ST with double stranded poly-A.** [A].Absorption spectrum of ST (3.05 µM) in absence (curve 1, black) and in presence of 301.09 µM ds poly-A (curve 2, red) in 10 mM CP buffer, pH 4.5 at 20°C. [B] Fluorescence spectrum of ST (5.0 µM) in absence (curve 1, black) and in presence of 510.50 µM ds poly-A (curve 2, red) in 10 mM CP buffer, pH 4.5 at 20°C. [C] Circular dichroic spectra of ds poly-A (100.0 µM) in absence (curve 1, black) and in presence of 55.60 µM ST (curve 2, red) in 10 mM CP buffer, pH 4.5 at 20°C.
(TIF)

Figure S2 **Denaturation and renaturation study.** Spectropolarimetric measurements on heating (red) and cooling (blue) of 100.0 µM of ss poly-A in absence (A) and in presence of ST (B, *D/P* = 0.53) in 10 mM CP buffer, pH 7.0. The arrows indicate the direction of heating (red) and cooling (blue) process. (C) Melting profile of 100.0 µM ds poly-A in absence (red) and in presence of ST (blue, *D/P* = 0.53) in10 mM CP buffer, pH 4.5.
(TIF)

Figure S3 **Salt dependence of CD spectral Changes.** Representative CD spectra resulting from the interaction of ss poly-A (100.0 µM) in (A) 5 mM [Na**^+^**] treated with 0, 4.28, 10.68, 21.32, 31.91, 42.44, 52.92 and 63.32 µM of ST (curves 1–8); (B) 10 mM [Na**^+^**] treated with 0, 4.28, 10.68, 21.32, 31.91, 42.44 and 52.92 µM of ST (curves 1–7); (C) 50 mM [Na**^+^**] treated with 0, 10.68, 31.91, 52.92 and 63.32 µM of ST (curves 1–5); and (D) 100 mM [Na**^+^**] treated with 0, 10.68, 31.91, 52.92 and 63.32 µM of ST (curves 1–5) in CP buffer, pH 7.0 at 20°C.
(TIF)
