# Supplementary figures and images for: Binding of Phenazinium Dye Safranin T to Polyriboadenylic Acid: Spectroscopic and Thermodynamic Study
Source: PLoS One. 2014 Feb 3;9(2):e87992. doi: 10.1371/journal.pone.0087992 (PMC3912202; doi:10.1371/journal.pone.0087992)

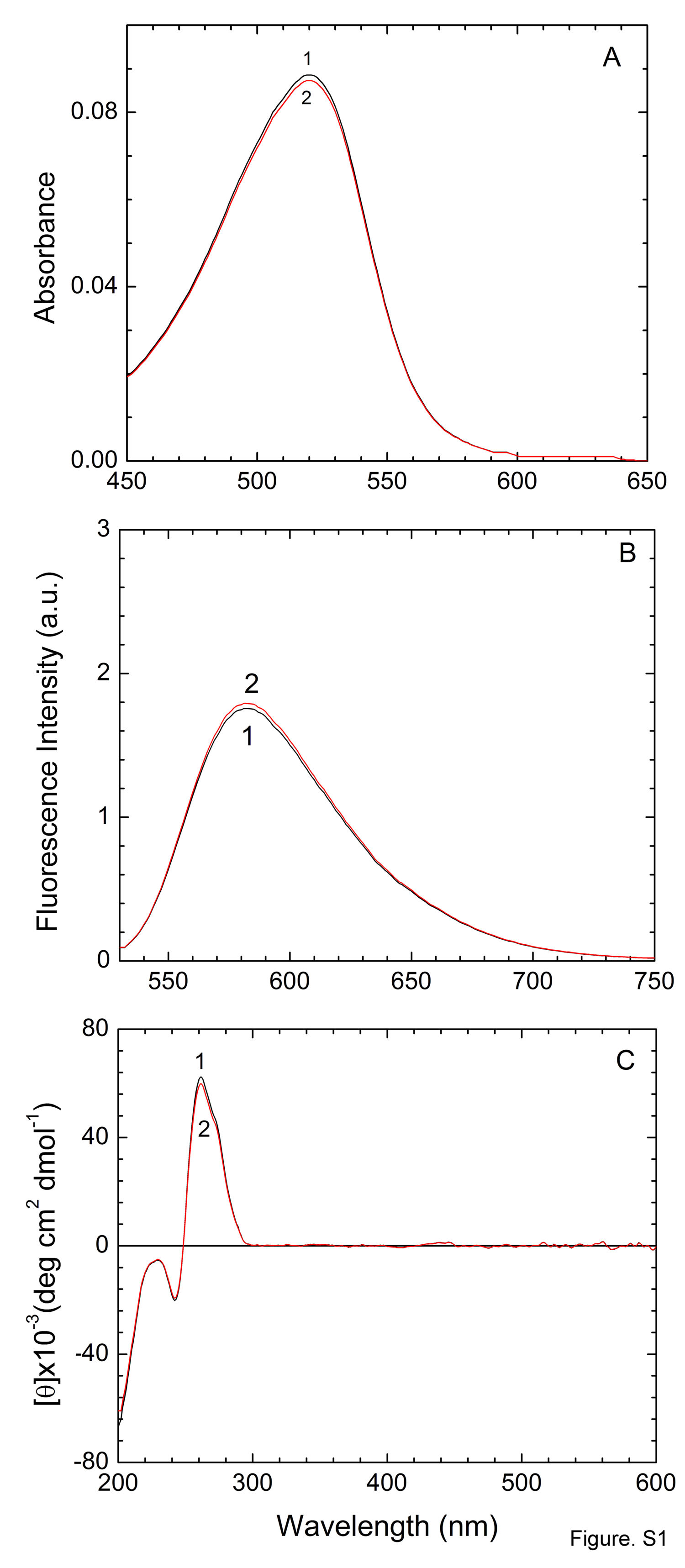

Supplement: Figure S1 — Characterization of interaction of ST with double stranded poly-A. [A].Absorption spectrum of ST (3.05 µM) in absence (curve 1, black) and in presence of 301.09 µM ds poly-A (curve 2, red) in 10 mM CP buffer, pH 4.5 at 20°C. [B] Fluorescence spectrum of ST (5.0 µM) in absence (curve 1, black) and in presence of 510.50 µM ds poly-A (curve 2, red) in 10 mM CP buffer, pH 4.5 at 20°C. [C] Circular dichroic spectra of ds poly-A (100.0 µM) in absence (curve 1, black) and in presence of 55.60 µM ST (curve 2, red) in 10 mM CP buffer, pH 4.5 at 20°C. (TIF) [file pone.0087992.s001.tif]

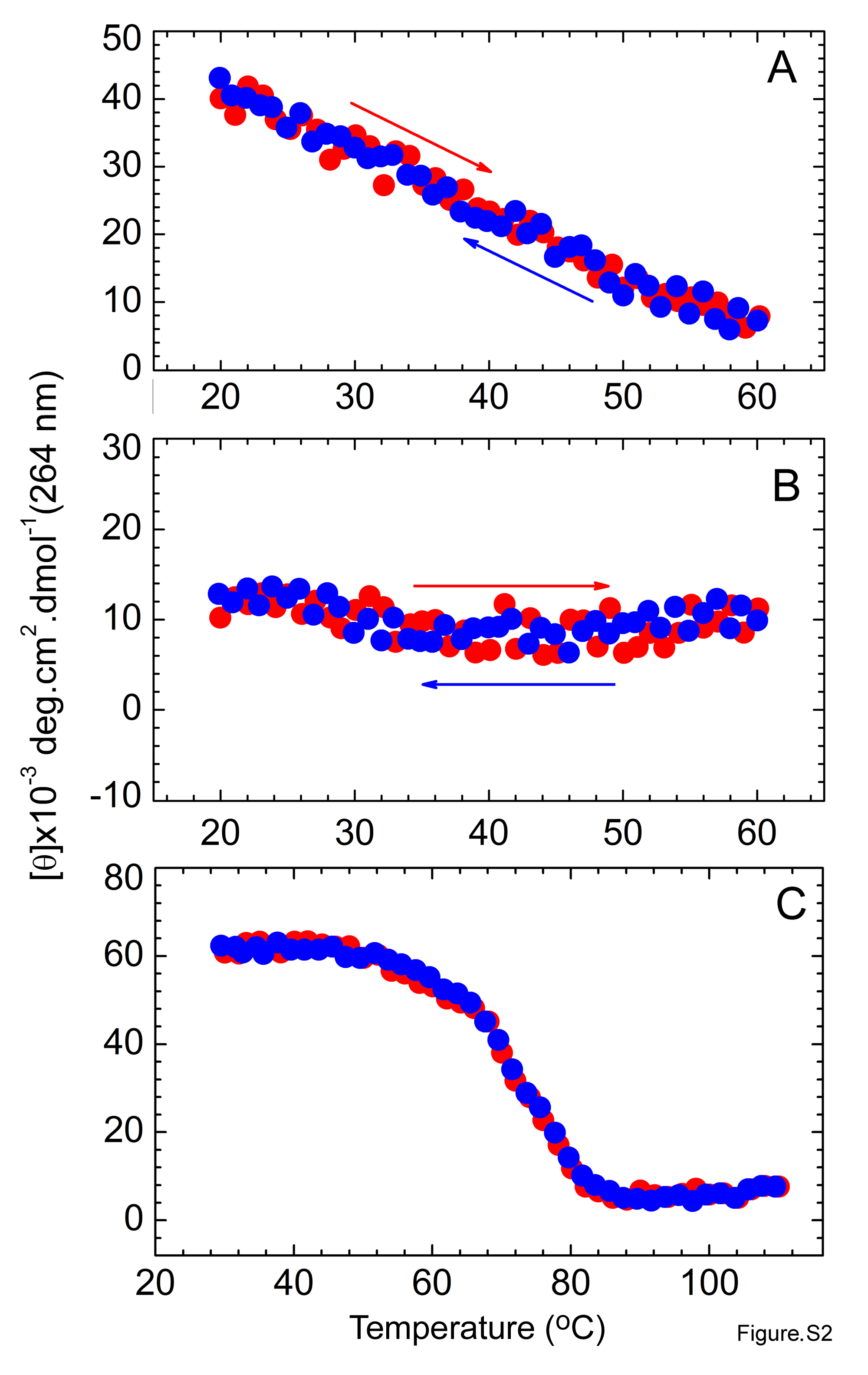

Supplement: Figure S2 — Denaturation and renaturation study. Spectropolarimetric measurements on heating (red) and cooling (blue) of 100.0 µM of ss poly-A in absence (A) and in presence of ST (B, D/P = 0.53) in 10 mM CP buffer, pH 7.0. The arrows indicate the direction of heating (red) and cooling (blue) process. (C) Melting profile of 100.0 µM ds poly-A in absence (red) and in presence of ST (blue, D/P = 0.53) in10 mM CP buffer, pH 4.5. (TIF) [file pone.0087992.s002.tif]

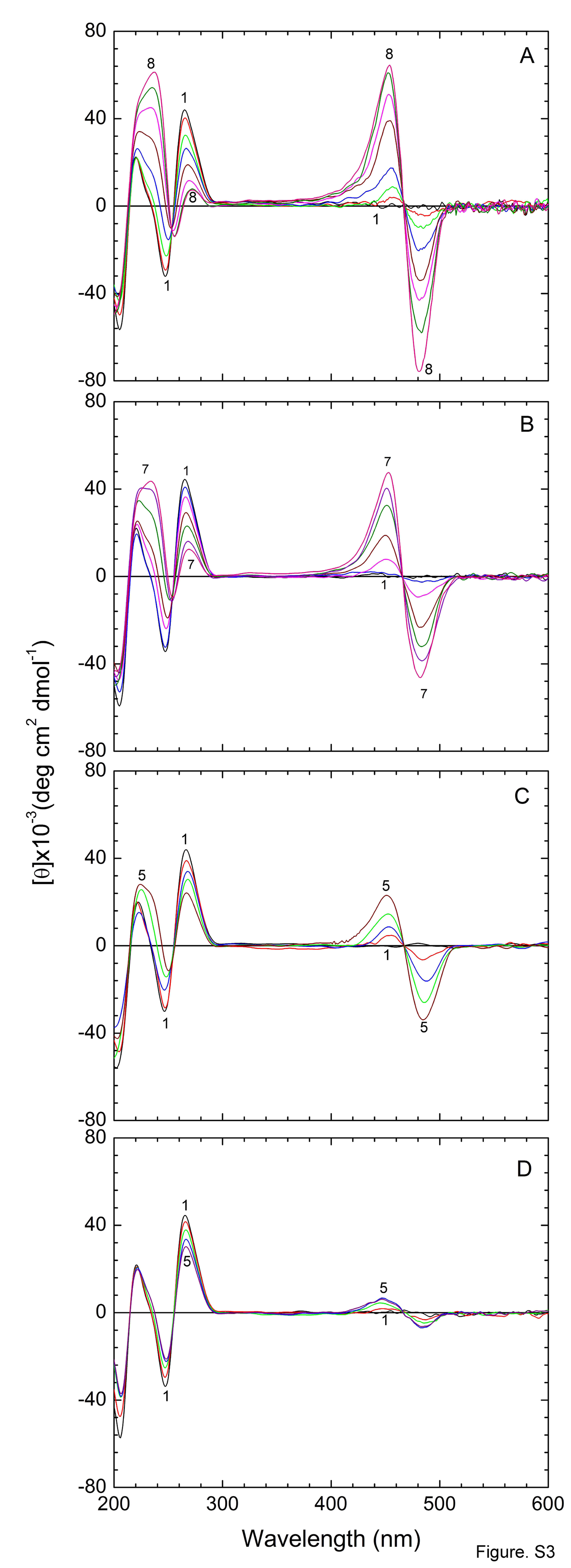

Supplement: Figure S3 — Salt dependence of CD spectral Changes. Representative CD spectra resulting from the interaction of ss poly-A (100.0 µM) in (A) 5 mM [Na+] treated with 0, 4.28, 10.68, 21.32, 31.91, 42.44, 52.92 and 63.32 µM of ST (curves 1–8); (B) 10 mM [Na+] treated with 0, 4.28, 10.68, 21.32, 31.91, 42.44 and 52.92 µM of ST (curves 1–7); (C) 50 mM [Na+] treated with 0, 10.68, 31.91, 52.92 and 63.32 µM of ST (curves 1–5); and (D) 100 mM [Na+] treated with 0, 10.68, 31.91, 52.92 and 63.32 µM of ST (curves 1–5) in CP buffer, pH 7.0 at 20°C. (TIF) [file pone.0087992.s003.tif]
